# Socioeconomic status impacts cognitive and socioemotional processes in healthy ageing

**DOI:** 10.1038/s41598-022-09580-4

**Published:** 2022-04-11

**Authors:** Joaquín Migeot, Mariela Calivar, Hugo Granchetti, Agustín Ibáñez, Sol Fittipaldi

**Affiliations:** 1 Latin American Brain Health Institute (BrainLat), Universidad Adolfo Ibáñez, Santiago de Chile, Chile; 2 Center for Social and Cognitive Neuroscience (CSCN), School of Psychology, Universidad Adolfo Ibáñez, Santiago de Chile, Chile; 3 Centro de Atención Primaria de la salud Zonda, Ministerio de Salud Pública de La Provincia de San Juan, San Juan, Argentina; 4 Facultad de Farmacia y Bioquímica, Universidad de Buenos Aires, Buenos Aires, Argentina; 5 Cognitive Neuroscience Center (CNC), Universidad de San Andrés, Victoria, Buenos Aires Argentina; 6 National Scientific and Technical Research Council (CONICET), Buenos Aires, Argentina; 7 Global Brain Health Institute, University of California San Francisco (UCSF), San Francisco, USA; 8 Trinity College Dublin (TCD), Dublin, Ireland; 9 Facultad de Psicología, Universidad Nacional de Córdoba, Córdoba, Argentina

**Keywords:** Human behaviour, Cognitive ageing

## Abstract

Socioeconomic status (SES) negatively impacts cognitive and executive functioning in older adults, yet its effects on socioemotional abilities have not been studied in this population. Also, evidence on neurocognitive processes associated with ageing primarily comes from Western, educated, industrialized, rich, and democratic (WEIRD) populations, hindering the generalization of findings to persons from upper-middle- and low‐middle‐income countries, such as those of Latin America. Here, we compared the performance of low- and high-SES older adults from Argentina in cognitive state, executive functions, social cognition (emotion recognition and theory of mind), and counter-empathic social emotions (envy and *Schadenfreude*; displeasure at others’ fortune and pleasure at others’ misfortune, respectively). Subsequently, we developed a path analysis to test the relationship among those variables in a theoretically plausible model and tested the main paths via multiple regression analyses. Relative to the high-SES group, low-SES older adults showed poorer performance on all assessed domains. Convergent evidence from covariance analysis, path analysis, and linear regressions suggested that low-SES impact on socioemotional processes was not primary but mediated by cognitive and executive impairment. These findings offer the first characterization of SES impacts on cognitive and socioemotional processes in a non-WEIRD population and have relevant equity-related implications for brain health.

## Introduction

Ageing is associated with a dysfunction in a variety of cognitive (e.g., attention, memory, language) and executive (e.g., working memory, inhibition) domains^1–3^, putatively related to reduced gray matter volume and connectivity among fronto-striatal, temporal, and parietal regions^4^. Evidence also suggests an ageing-associated dysfunction in social cognition abilities, including emotion recognition^5^ and theory of mind (ToM; the capacity to reason about others’ mental sates)^6–8^, which would be mediated by cognitive state^9,10^ and executive functions, partially related with task demands^11,12^. In turn, social cognition impairment could affect the experience of complex social emotions (i.e., those that are triggered in the presence of others), such as counter-empathic emotions, since they involve emotion recognition and perspective-taking processes^13^. Taken together, ageing-associated dysfunction in cognitive and executive processes would reduce social cognition abilities, which would negatively impact social emotions.

Socioeconomic status (SES) is a construct that describes the social (e.g., formal education, work prestige) and economic (e.g., material goods, monetary income) resources of an individual, and is one of the main factors from which life outcomes are derived^14^. Low-SES older adults have less access to cognitively stimulating environments and cultural resources, which hinders their cognitive (e.g., memory)^15^ and executive (e.g., working memory)^16^ functioning. Cognitive and executive functions deteriorate at different rates across the lifespan (i.e., with executive domains showing the earliest and fastest decline^17–20^), dissociate in early stages of neurodegenerative conditions^21^, and can respond differently to interventions^22,23^. Thus, they might be differentially impacted by SES. In support of this claim, recent evidence shows that, while childhood SES disadvantages can negatively impact cognitive functioning in old age, high childhood SES is associated with a faster decline in verbal fluency (an ability closely related to executive functions)^24^.

While the SES impact on social cognition has not been formally tested in the elderly, there is evidence of a positive relationship between education (one component of SES) and ToM abilities in this population^10^. Given the strong effect of education on cognitive^25,26^ and executive^26,27^ functions, and the reliance of social cognition on those processes^9–12^, a mediation effect would be expected. Indeed, low-SES middle-aged adults show preserved—or even heightened—socioemotional skills such as emotion recognition, empathy, and cooperation, putatively related to its adaptative value in adverse contexts^28–30^. Then, social cognition dysfunction in low-SES older adults could be plausibly explained by primary cognitive and executive impairments. In the same vein, as the experience of social emotions would depend on social cognition processes^13^, SES would affect it indirectly. In sum, it is well established that low-SES negatively affects cognitive state and executive functions in older adults, being its impact on socioemotional processes yet to be determined in this population.

Most evidence on neurocognitive processes associated with ageing comes from Western, educated, industrialized, rich, democratic (WEIRD) populations^31^, limiting the generalizability of findings to persons living in upper-middle- and low‐middle‐income countries (UMIC and LMIC, respectively), such as those of Latin America. Specifically, there is a paucity of research on ageing-associated cognitive and socioemotional dysfunction and the modulator role of SES on those domains in LMIC regions. In particular, Latin America is one of the most socioeconomically inequal regions of the world^32^, which is reflected in unique cognitive and socioemotional profiles^33^. Characterizing how SES modulates regionally situated cognitive ageing may offer relevant information in terms of global approaches to brain health and inclusive preventive care practices.

Against this background, this work aims to study the effect of SES on cognitive and socioemotional processes in healthy older adults from Latin America. First, we compared cognitive state, executive functions, social cognition, and social emotions (envy and *Schadenfreude*; displeasure at others’ fortune and pleasure at others’ misfortune, respectively^34^) among low- and high-SES older adults’ samples from Argentina. Then, we developed a path analysis to test the relationship among those variables in a theoretically plausible model. This technique, by comparing the correlational patterns of actual data with those proposed in a model, allows to estimate the direct and indirect pathways by which variables influence each other^35^. Based on the existing evidence, we constructed a model in which (a) SES has direct effects on cognitive state^15^ and executive functions^16^; (b) SES has an indirect effect on social cognition, mediated by cognitive state^10,25,26^ and executive functions^26,27^; and (c) SES has a total effect on social emotions. Finally, the robustness of individual predictions was tested using linear regression analyses. We hypothesize that (1) low-SES group would show lower scores than their high-SES counterparts on all assessed domains, (2) impaired social cognition in the low-SES group would be mediated by cognitive state and executive functions deficits, (3) impaired social emotions in the low-SES group would be mediated by social cognition, and (4) our models would predict a significant portion of the variance of observed data and show adequate fit indices.

## Methods

### Participants

Fifty-eight older adults were enrolled in this study. Thirty low-SES participants were recruited from ‘Zonda’ primary care center at San Juan province, Argentina. Inclusion criteria were to be ≥ 55 years old and qualify as low- or medium-low-SES level according to the European Society for Opinion and Marketing Research (ESOMAR) questionnaire^36^, as detailed below. Twenty-eight high-SES participants were recruited from nearby regions though convenience sampling to match with low-SES participants in sex, age, and current occupational status (active and retired). They had to classify as very-high-, high-, or medium-high-SES level according to the ESOMAR (see below). No participant was unemployed at the moment of the assessment. Also, no participant presented history of neurological or psychiatric disorders, nor substance abuse, as assessed by a semi-structured clinical interview.

The ESOMAR^36^ is a useful tool to measure SES in Latin American population^37^. It is based on the assessment of the educational level (last grade accomplished, ranging from primary school to postgraduate studies) and occupation (main profession or job, ranging from informal jobs to general manager of large companies) of the head of the household. In the case of retired participants, a battery evaluating the possession of goods (e.g., car, computer, microwave) is used. The ESOMAR provides six levels to classify participants’ SES: A = very high, B = high, C_a_ = medium–high, C_b_ = medium, D = medium–low, and E = low. Here, we based on Celis-Morales et al.^37^ and Lizana et al.^38^ to merge levels D (*n* = 18) and E (*n* = 12) into the low-SES group (*n* = 30), and levels B (*n* = 15) and C_a_ (*n* = 13) into the high-SES group (*n* = 28). Individuals pertaining to C_b_ level were excluded from the sample and none of the participants was qualified as level A. See Table 1 for groups’ data and statistics and Supplementary Table S1 for specific ESOMAR dimensions’ assessment.

**Table 1:** Groups’ demographics and experimental variables.

Variable	Low-SES (*n* = 30)	High-SES (*n* = 28)	Statistics
**Sex**			
F	15	16	*χ*2(1, *n* = 58) = 0.30, *p* = 0.59
M	15	12	
Age	63.63 (7.60)	63.93 (7.61)	*t*(56) = 0.15, *p* = 0.88
Years of education	7.20 (2.89)	15.64 (3.34)	*t*(56) = 10.32, *p* < 0.001, Cohen’s *d* = 2.71
**Occupational status**			
Active	21	19	*χ*2(1, *n* = 58) = 0.03, *p* = 0.86
Retired	9	9	
**Dominant hand**			
Right	28	26	χ2(1, *n* = 58) = 0.01, *p* = 0.94
Left	2	2	
Depressive symptoms (BDI-II score)	11.5 (10.43)	6.62 (5.61)	*t*(50) = 2.10, *p* = 0.04, Cohen’s *d* = 0.58
Cognitive state (ACE-III total score)	79.20 (8.10)	91.18 (3.78)	*t*(56) = 7.13, *p* < 0.001, Cohen’s *d* = 1.87; *F*(1, 49) = 37.72, *p* < 0.001, *η*^2^ = 0.44
Executive functions (IFS total score)	19.15 (3.55)	23.17 (2.13)	*t*(51) = 4.81, *p* < 0.001, Cohen’s *d* = 1.33; *F*(1, 44) = 17.26, *p* < 0.001, *η*^2^ = 0.28
Social cognition (Mini-SEA total score)	23.59 (3.06)	26.29 (1.85)	*t*(49) = 3.86, *p* < 0.001, Cohen’s *d* = 1.10; *F*(1, 40) = 0.17, *p* = 0.68, *η*^2^ = 0.01
Social emotion: envy score	4.46 (2.35)	5.53 (1.54)	*t*(56) = 2.03, *p* = 0.04, Cohen’s *d* = 0.53; *F*(1, 39) = 3.01, *p* = 0.09, *η*^2^ = 0.07
Social emotion: *Schadenfreude* score	0.75 (3.01)	2.96 (2.72)	*t*(56) = 2.93, *p* = 0.01, Cohen’s *d* = 0.77; *F*(1, 39) = 2.44, *p* = 0.13, *η*^2^ = 0.06

Data are presented as mean (*SD*) except for sex, occupational status, and handedness. Categorical variables were analyzed with Chi square tests. Continuous variables were analyzed with Student’s independent-samples *t*-tests and ANCOVA (see details in “Methods” section). ACE-III: Addenbrooke’s Cognitive Examination-III; BDI-II: Beck Depression Inventory-II; IFS: INECO Frontal Screening; Mini-SEA: Mini-Social Cognition and Emotional Assessment; SES: socioeconomic status.

Groups differed in years of education (Table 1), which is expected since educational level is part of the SES definition and assessment. Also, low-SES participants presented higher depressive symptoms, as measured with the Beck Depression Inventory-II (BDI-II)^39^, a self-report questionnaire that tracks cognitive, affective, and somatic manifestations of depression (Table 1). Evidence shows that low-SES is related with the development of affective symptoms^40^. Thus, BDI-II score was included as covariate of no interest in all analyses.

### Assessment protocol

#### Cognitive state

Cognitive state was assessed with the Addenbrooke’s Cognitive Examination-III (ACE-III)^41^, which evaluates five cognitive domains: (1) orientation and attention, (2) memory, (3) verbal fluency, (4) language, and (5) visuospatial skills. The ACE-III total score ranges from 0 to 100 (cut-off: 86), with higher scores indicating better performance. The ACE-III has proved useful in detecting cognitive impairment in healthy older adults^42^.

#### Executive functions

Executive functions were assessed with the INECO Frontal Screening (IFS)^43^, a brief screening tool that taps three executive functions: response inhibition and set shifting, working memory, and abstraction capacity. The IFS total score ranges from 0 to 30, with higher scores indicating better executive performance. The IFS has good sensitivity to evaluate frontal-executive dysfunction in older adults^44^.

#### Social cognition

The Mini-Social Cognition and Emotional Assessment (Mini-SEA)^45^ was used to asses two social cognition domains: facial emotion recognition and ToM. In the facial emotion recognition subtest, participants are required to select the emotion being expressed by an individual's face among the following options: sadness, disgust, anger, fear, happiness, surprise, or neutral. In total, 35 items are displayed, giving 1 point for each correct response. ToM is evaluated by the Faux Pas subtest, in which short stories are presented to participants, who must detect social transgressions and infer the feelings and intentions of the stories’ characters. It consists of 10 items, and the total score ranges from 0 to 40. To obtain the overall Mini-SEA score, both facial emotion recognition and Faux Pas total scores are converted to a score out of 15 and then summed, ranging from 0 to 30, with higher scores indicating better performance. This test has been previously employed to evaluate social cognition among older adults^46,47^.

#### Social emotions

Envy and *Schadenfreude* were assessed through an experimental paradigm based on Takahashi et al.^34^, adapted to Spanish language and validated in Latin American populations^48–53^. The task consists of the presentation of 40 fictional situations designed to evoke unpleasant (envy, e.g., ‘He won the lottery and he is a millionaire’, *n* = 15) or pleasant (*Schadenfreude*, e.g., ‘He was excluded from his friends’ group because he is a liar’, *n* = 15) emotions, as well as emotionally neutral (e.g., ‘He turned off the light’) situations included separately for envy (*n* = 5) and *Schadenfreude* (*n* = 5). First, participants are presented with a real-life photograph and a description of two characters matched to the participant in terms of age and sex. Subsequently, the situations for each type of emotion are described involving the characters individually. Envy- and *Schadenfreude*-eliciting situations are presented in two different blocks (envy first), alternating with the corresponding neutral situations. In both blocks, after reading each situation, participants are asked to report the intensity of their ‘displeasure’ (for the envy block) or ‘pleasure’ (for the Schadenfreude block) on a 1 (low emotional intensity) to 9 (high emotional intensity) Likert scale. To control for basic understanding of the task structure, weighted variables for envy and Schadenfreude are computed by subtracting the neutral scenarios’ score to each emotion’s score. Hereafter, envy weighted and Schadenfreude weighted variables are referred as envy and Schadenfreude, respectively.

As previously reported^48,51–54^, envy- and *Schadenfreude*-eliciting situations of the task consistently produce higher ‘displeasure’ and ‘pleasure’ ratings than neutral scenarios. Furthermore, a validation study confirmed that said situations were specific in evoking envy and *Schadenfreude* compared to other socio-moral emotions such as pride and guilt^50^. The task has been employed in samples of older adults from Latin America^50,51^, successfully evoking envy and *Schadenfreude*^50,51^, and capturing its canonical neuroanatomical basis^50^ (i.e., the anterior cingulate cortex for envy^34^ and the striatum and posterior regions for *Schadenfreude*^34,55^).

### Data analyses

Low- and high-SES groups’ performance on cognitive state (ACE-III), executive functions (IFS), social cognition (Mini-SEA), and social emotions (envy and *Schadenfreude*) were compared using Student’s independent-samples *t*-tests. Then, we performed ANCOVA tests to control for the effect of confounding variables on each domain. Specifically, we adjusted for the effect of (1) depressive symptoms (BDI score) on cognitive state and executive functions (ACE-III and IFS)^40^, (2) depressive symptoms, cognitive state, and executive functions on social cognition (Mini-SEA score)^9,11^, and (3) depressive symptoms, cognitive state, executive functions, and social cognition on social emotions (envy and *Schadenfreude* scores)^13^.

Subsequently, path analysis models were performed. This analysis strategy allows to empirically test theorized relationships among observed variables (i.e., measured by one single indicator) through direct effects (i.e., causal effect of one variable over another), indirect effects (i.e., causal effect of one variable over another mediated by one or more variables), and total effects (i.e., causal effect of one variable over another summing all direct and indirect effects among those variables), along with fit indices of the causal relationships draw in the model^35^. Before performing a path analysis, some requirements must be accomplished, such as the sample size in relation to the number of paths included in the model, directionality of presumed causal relationships, score reliability of the measures employed, and normality of the variables^56,57^ (see Supplementary material 1 for a description of the fulfillments of those requirements in our models).

We performed two independent path models (one with envy and another with *Schadenfreude* as final outcomes) including the same number of predictor variables and paths. Thus, the models predicted: SES ⟶ cognitive state/executive functions ⟶ social cognition ⟶ social emotions (model 1: envy; model 2: *Schadenfreude*). Direct, indirect, and total effects were assessed through 10,000 bootstrap iterations as per Mallinckrodt et al.^58^, from which confidence intervals at 95% and 97.5% were obtained. Here, SES was decomposed into four levels to improve measure variance^38^ (see Supplementary material 2 for quadripartite group formation description and Supplementary Table S2 for demographic matching). To handle missing data (Table S3) we employed a strategy of data imputation by regression (Supplementary material 3). We aimed to reach the *N*:*q* ratio of 10:1 to achieve statistical precision and power^56,57^, as our sample size (*n* = 58) marginally equals ten times the number of paths included in our models (*q* = 6). The fit indices RMSEA (≤ 0.05 indicating good fit), SRMR (< 0.08 indicating good fit), NFI (> 95 indicating good fit), GFI (> 95 indicating good fit), and CFI (> 95 indicating good fit) were employed to test the hypothesized models’ fit^56,59^. A null path analysis model with the same number of paths was performed to contrast its fit with that of the hypothesized models. It consisted in a direct path from SES to all variables, from cognitive state to executive functions, and from social cognition to envy.

Finally, given our modest sample size, we performed simple and multiple linear regression analyses to verify the direct effects included in our path models, namely the prediction of (1) SES ⟶ cognitive state, (2) SES ⟶ executive functions, (3) cognitive state ⟶ executive functions, (4) cognitive state and executive functions ⟶ social cognition, (5) social cognition ⟶ envy, and (6) social cognition ⟶ *Schadenfreude*.

Two-tailed *t*-test, ANCOVA, and linear regression analyses were performed using the Statistical Package for Social Sciences (SPSS, version 25.0) software. Effect sizes were calculated through Cohen’s *d* for *t*-tests, and partial eta squared (*η*^2^) ratio for ANCOVA analyses. Path analyses were performed on Analysis of Moment Structure (AMOS, version 25.0) software. The alpha threshold was set at 0.05 for all analyses.

## Ethics declarations

The study was approved by the ethics committee of the Institute of Cognitive Neurology in Buenos Aires, Argentina. All experiments were performed following guidelines and regulations of the Declaration of Helsinki. Signed informed consent was obtained from all participants.

## Results

### Between-group’s differences

#### Cognitive state

**Figure 1:**
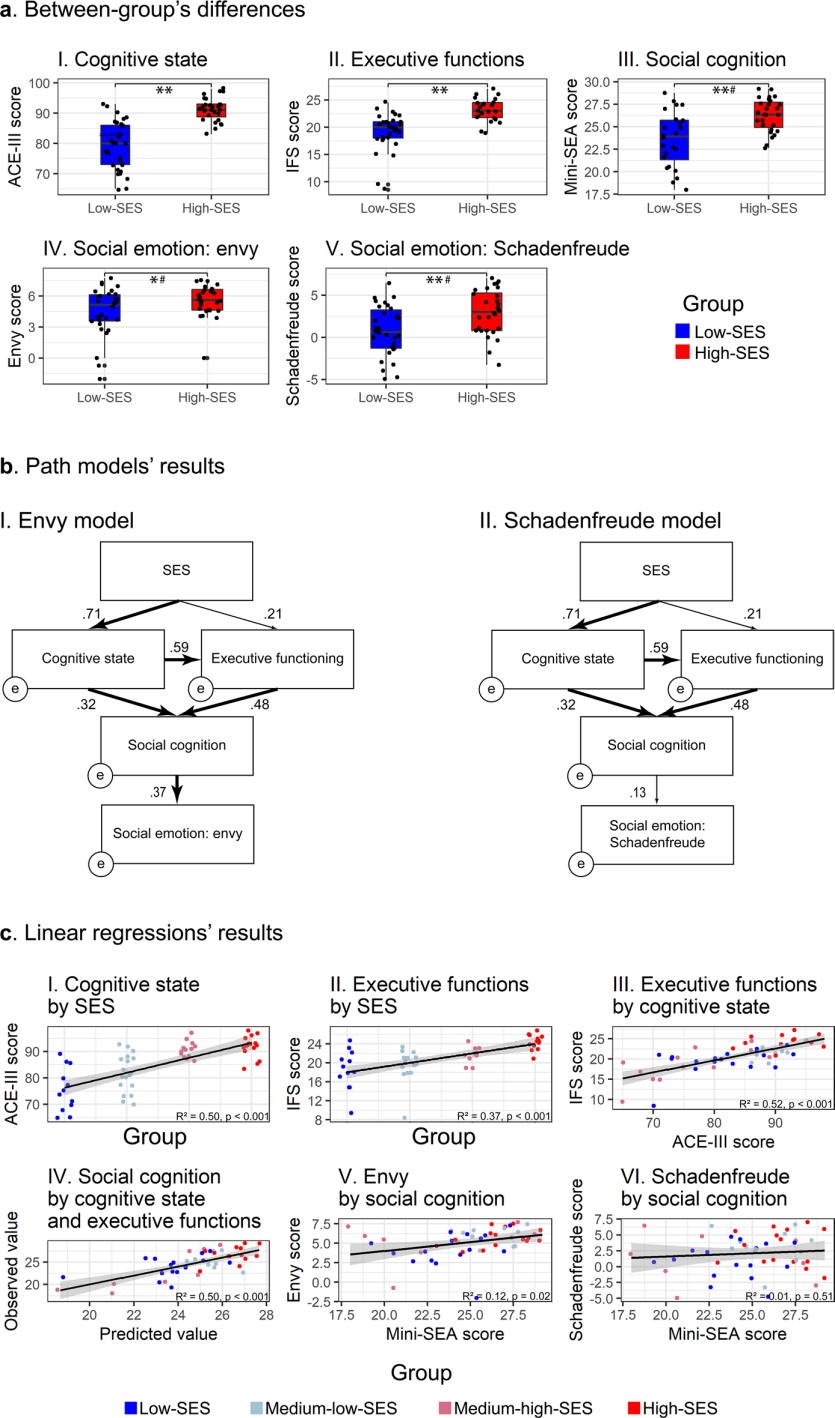
Results. (**a**) Data distribution and performance differences between low- and high-SES groups on cognitive state (**I**), executive functions (**II**), social cognition (**III**), and social emotions; envy (**IV**) and *Schadenfreude* (**V**). Boxplots display the median, interquartile range, and range of each variable. *p < 0.05, **p < 0.001, ^#^*p* > 0.05 after controlling for intervening variables (see “Methods” section). (**b**) Path analysis models and standardized path coefficients weights for envy (**I**) and *Schadenfreude* (**II**) as outcome social emotions. Bold arrows depict statistically significant paths. (**c**) SES predicts cognitive state (**I**) and executive functions (**II**). Cognitive state predicts executive functions (**III**). Cognitive state and executive functions predict social cognition (**IV**). Social cognition predicts envy (**V**) but not *Schadenfreude* (**VI**). All linear regressions’ results are statistically significant (*p* < 0.05), except for (**VI**). ACE-III: Addenbrooke’s Cognitive Examination-III; IFS: INECO Frontal Screening; Mini-SEA: Mini-Social Cognition and Emotional Assessment; SES: socioeconomic status.

Low-SES group showed worse performance than high-SES group on cognitive state. Results remained statistically significant when controlling for depressive symptoms (Table 1, Fig. 1a).

#### Executive functions results

Low-SES group exhibited worse performance than high-SES group on executive functions. Results remained statistically significant when controlling for depressive symptoms (Table 1, Fig. 1a).

#### Social cognition

Low-SES group presented lower performance than high-SES group on social cognition. However, the difference disappeared when controlling for depressive symptoms, cognitive status, and executive functions scores altogether (Table 1, Fig. 1a), suggesting an influence of those domains on social cognition.

#### Social emotions

Low-SES group reported lower intensity in their experience of social emotions (envy and *Schadenfreude*) than high-SES group. Nevertheless, results disappear when covarying by depressive symptoms, cognitive state, executive functions, and social cognition scores altogether (Table 1, Fig. 1a).

### Path analysis

Two path analysis models were developed to estimate the direct and indirect pathways by which SES (ESOMAR) influences cognitive state (ACE-III), executive functions (IFS), social cognition (Mini-SEA), and social emotions (envy and *Schadenfreude*). A model was performed for each social emotion (Fig. 1b).

When including envy as the outcome of social emotions, the path analysis showed a very good fit (*χ*^2^ (4, *n* = 58) = 2.39, *p* = 0.66), as well as values of fit indices (RMSEA = 0.00 (0.00–0.16); SRMR = 0.03; NFI = 0.98; GFI = 0.98; CFI = 1.00).

SES had a direct effect on cognitive state (*p* < 0.001), but its effect on executive functions failed to reach significance (*p* = 0.10). Cognitive state exhibited a direct effect on executive functions (*p* < 0.001), and both variables showed a direct effect on social cognition (cognitive state: *p* = 0.01; executive functions: *p* < 0.001). For its part, social cognition showed a direct effect on envy (*p* = 0.01). On the other hand, SES had an indirect effect on social cognition mediated by cognitive state (*p* = 0.02), but its indirect effect on social cognition was not significant when mediated by executive functions (*p* = 0.15). Additionally, SES indirectly predicted social cognition when jointly including cognitive state and executive functions as mediator variables (*p* < 0.001). Finally, SES displayed a significant total effect on envy (*p* = 0.01) (see Supplementary Table S4 for standardized and non-standardized direct, indirect, and total effects’ coefficients statistics, and Supplementary Table S5 for confidence intervals).

When including *Schadenfreude* as the outcome of social emotions, the path analysis model exhibited a smaller but acceptable fit (*χ*^2^ (4, *n* = 58) = 6.48, *p* = 0.17) and values of fit indices (RMSEA = 0.10 (0.00–0.25); SRMR = 0.08; NFI = 0.95; GFI = 0.96; CFI = 0.98). As in the envy model, SES had a direct effect on cognitive state (*p* < 0.001), but not on executive functions (*p* = 0.10). Also, cognitive state showed a direct effect on executive functions (*p* < 0.001), and both exhibited a direct effect on social cognition (cognitive state: *p* = 0.01; executive functions: *p* < 0.001). In contrast to the envy model, social cognition did not show a direct effect on *Schadenfreude* (*p* = 0.32). SES exhibited an indirect effect on social cognition mediated by cognitive state (*p* = 0.02), but its effect on social cognition failed to reach significance when including executive functions as the mediator variable (*p* = 0.15). Moreover, SES predicted social cognition mediated by cognitive state and executive functions together (*p* < 0.001). Finally, SES’ total effect on *Schadenfreude* was not significant (*p* = 0.34) (see Supplementary Table S6 for standardized and non-standardized direct, indirect and total effects’ coefficients statistics and Supplementary Table S7 for confidence intervals).

In contrast, the random (null) path analysis model (SES ⟶ all variables, cognitive state ⟶ executive functions, and social cognition ⟶ envy) yielded no relevant effects in terms of fit (*χ*^2^ (4, *n* = 58) = 29.15, *p* < 0.001), as well as values of fit indices (RMSEA = 0.33 (0.23 – 0.45); SRMR = 0.12; NFI = 0.80; GFI = 0.86; CFI = 0.81).

### Linear regressions’ results

Simple linear regression models showed that SES (ESOMAR) predicted cognitive state (ACE-III) (*F*(1, 56) = 56.63, *p* < 0.001, *R*^2^ = 0.50) and executive functions (IFS) (*F*(1, 51) = 30.26, *p* < 0.001, *R*^2^ = 0.37). Additionally, cognitive state predicted executive functions (*F*(1, 51) = 54.86, *p* < 0.001, *R*^2^ = 0.52). A multiple linear regression model revealed that cognitive state and executive functions predicted social cognition (Mini-SEA) (*F*(2, 43) = 21.52, *p* < 0.001, *R*^2^ = 0.50), with executive functions being the single significant predictor (*β* = 0.44, *p* = 0.01; cognitive state: *β* = 0.31, *p* = 0.06). Finally, social cognition predicted envy (*F*(1, 49) = 6.40, *p* = 0.02, *R*^2^ = 0.12), but did not predicted *Schadenfreude* (*F*(1, 49) = 0.44, *p* = 0.51, *R*^2^ = 0.01) (Fig. 1c).

## Discussion

To our knowledge, this is the first study in characterizing SES impacts on cognitive and socioemotional processes in healthy older adults from Latin America. As expected, in line with evidence from WEIRD populations^15,16^, we found that low-SES is associated with diminished cognitive state and executive functions. Furthermore, we showed for the first time that low-SES has an indirect negative impact on social cognition (i.e., emotion recognition and ToM) and on a counter-empathic social emotion (envy) in ageing, mediated by affective, cognitive, and executive dysfunction. Results provide new insights for global approaches to cognition and have relevant equity-related implications.

Disadvantages in low-SES older adults compared to their high-SES counterparts were more pronounced for cognitive and executive domains than for socioemotional ones, as reflected by larger effect sizes. Moreover, between-group differences in cognitive state and executive functions persisted after controlling for depressive symptoms. In turn, between-group differences in social cognition and social emotions disappeared when controlling for depression, cognitive state, and executive functioning, indicating a *non-sui generis* effect of SES on socioemotional processes. While there is a lack of research in ageing, this pattern of results is consistent with evidence from children and middle-aged adults. Children’s studies find higher effect of SES on cognitive skills and academic achievement than on socioemotional competence^60,61^. Arguably, ‘cold’ cognitive processes would be more vulnerable to unfavorable contextual factors associated with low-SES, such as diminished access to cognitively stimulating activities, education, and early life nutrition^14,62^. Convergently, studies on middle-aged adults suggest that socioemotional skills, such as dispositional compassion and orientation to engage with others^28^, empathic accuracy, and emotion recognition^29,30^, are spared among those with low-SES given its adaptative function in facilitating the interdependence with others to cope with adversity^63^. Our results extend these interpretations to older adults by revealing that socioemotional decrease associated with low-SES in this population would be dependent on cognitive and executive impairment.

Depressive symptoms did not affect cognitive state and executive functions and showed a partial effect on social cognition and social emotions, as shown by ANCOVAs’ results. Low-SES older adults tend to show higher prevalence of depressive symptoms^64–66^, which could hinder their cognitive^67^ and socioemotional^68,69^ functioning. Chronic stressors exposure, such as financial needs^70,71^, has been related to hippocampal and amygdala volume reduction and aberrant prefrontal cortex activity^72,73^, and these regions are crucially involved in memory, emotion processing, executive functions, and social behavior. However, our results suggest that impairments associated with low-SES in older adults are not entirely explained by depression. Potentially, the experience of stress has mediator role, which should be addressed in future studies.

Path analyses’ results supported the proposed theoretical model. SES directly predicted cognitive state, which is consistent with previous evidence depicting its effects in older adults^15,16^. The direct effect of SES on executive functions failed to reach significance, suggesting executive impairment would mainly depend on cognitive state^10^. Furthermore, SES indirectly predicted social cognition mediated by cognitive state and executive functions, with executive functions emerging as the single significant predictor when controlling for the variance of cognitive state in multiple regression analysis. This is in line with previous works showing that social cognition abilities—such the inference of others’ emotions and mental states—are supported by executive functions, particularly the capacity to inhibit the own perspective to adopt that of others (i.e., cognitive flexibility)^10,12,74^. In sum, our results suggest that low-SES impairs cognitive state, which results in less resources to executive functions, which in turn would fail to support social cognition. This seems to be the first evidence in elucidating a potential mechanism by which SES impacts social cognition in older adults.

Path models also revealed that SES had a total effect on envy but not on *Schadenfreude*. Relatedly, social cognition predicted envy but not *Schadenfreude*. These differential impacts might be explained by the distinct involvement of neurocognitive processes in each emotion. Envy is a painful emotion that involves self-concept threat, cognitive dissonance representation, conflict monitoring, and inhibitory control (to override negative actions’ tendencies)^13,34,50^. These processes converge in the anterior cingulate cortex, the main neural basis of envy^34^, and one of the core hubs of executive control^75^. In contrast, *Schadenfreude* is a pleasant emotion, characterized by reward processing in association with ventral striatum activity^34^ and volume^49^, putatively implying a more basic representational process and less cognitive load than envy. Indeed, older adults tend to show reduced intensity in their experience of envy^76^. Therefore, given the low-SES impact on social cognition via executive functions, and the closer link between executive functions and envy (but not *Schadenfreude*), it is plausible that SES would selectively modulate envy. These findings are the first to depict a differentiated functional dependency of social emotions in older adults, with selective susceptibility to SES. Future neuroimaging studies should assess the neural basis of SES modulations on envy and *Schadenfreude* to confirm this claim.

Our findings have implications to better understand inequity influences on cognition, to differentiate normative from pathological ageing through a socioeconomically situated approach, and to develop regionally relevant practices to promote brain health. Higher educationaland occupational attainments may attenuate not only cognitive but also socioemotional dysfunction associated with brain ageing. Considering the rising ageing and dementia rates in Latin American population^77^, alongside the worrying socioeconomic inequality of the region^78^, our results constitute a call for action. There is an urgent need to develop locally appropriate preventive practices. For instance, low-SES older adults may require earlier care due to their pronounced dysfunction.

Some limitations and future directions of this work must be acknowledged. First, our SES estimation is based on educational level and main occupation, or, alternatively, level of possessions, excluding other relevant determinants such as income or neighborhood quality^14^. Future studies should explore the potential differential impact of SES components on cognitive and socioemotional processes as well as outcomes of their fluctuations over time (e.g., a person might face transient economic privations associated with intermittent crisis). Interestingly, recent evidence suggests that, while education is a strong predictor of general knowledge (a component of crystallized intelligence), SES (measured as subjective social status^79^) is not^80^. Thus, life-long educational trajectories may modulate the effect of early SES disadvantages on cognition. Longitudinal studies are necessary to empirically test this possibility and to examine causal effects.

Second, our sample size was modest, marginally satisfying the requirements to achieve statistical precision and power in path analyses and limiting generalization of findings. This constraint is particularly relevant when interpreting goodness of fit indices such as *χ*^2^, as this coefficient directly increases in relation to sample size (resulting in diminished *p*-values)^81^. Thus, a small sample size can lead to a small *χ*^2^ and an artefactually inflated *p*-value, affecting the interpretation of the model’s fit^81^. Yet, we employed multiple goodness of fit indices robust to sample size variations (e.g., CFI^82^), and, as expected, the null path model with the same sample size showed poorer fit, allowing to trust the *χ*^2^ index. Also, the paths included in our models were validated by strong linear regressions’ results and our sample size was comparable to other studies in the field^83,84^.

Third, we did not evaluate specific cognitive, executive, and social cognition subdomains (e.g., memory, language, working memory, emotion recognition) in our models nor tested other pathways to social emotions than social cognition because it was beyond the scope of the study –and our sample size would have been insufficient. Our models were hypotheses-driven (as stated in the Introduction) and the conceptual split of the selected domains (e.g., cognitive—executive functions) is grounded on relevant evidence^1–3,17–23^. Moreover, the inclusion of global scores for cognitive state, executive functions, and social cognition is a common practice in path analysis models^85–87^. Nonetheless, exploratory results including ACE-III subdomains instead of the total score in the models were consistent with our main interpretations (Supplementary material 4). Briefly, all models showed good fit indices (Supplementary Tables S8 and S9) and, while verbal fluency and language predicted social cognition, orientation and attention, memory, and visuospatial skills did not (Supplementary Tables S10 to S19, and Supplementary Fig. S1 and S2), as expected when considering task demands (see additional discussion in Supplementary material 4). In any case, future studies should implement a larger sample size to confirm the robustness of our findings and test specific hypotheses regarding subdomains and pathways.

Relatedly, our assessment protocol did not include interpersonal perception measures (e.g., supportive social networks), preventing a comprehensive view of SES’ effects on cognitive and socioemotional performance. For instance, loneliness is associated with lower cognitive functioning in older adults^88^ and impairs socioemotional processes by heightening the sensitivity to social threats^89^. Future studies should combine neuropsychological, interpersonal perception, and social functioning measures to allow testing potential mediators of SES on cognition.

Fourth, while we focused on envy and *Schadenfreude*, it would also be useful to address other social emotions such as shame and pride, which have proven relevant for older adults’ quality of life^90^. Finally, future works should compare WEIRD and non-WEIRD older adults in order to empirically test differences between diverse and underrepresented populations.

In conclusion, low-SES seems to primarily affect cognitive state and, indirectly (through executive functions), socioemotional processes in healthy older adults. Moreover, social emotions would be differentially compromised by SES, depending on their underlying neurocognitive functions. Our results are relevant to better understand the effect of inequity on brain health and to advance the development of timely preventive care actions to overcome present and future challenges of ageing in LMIC and across Latin America.

## Supplementary Information

Supplementary Information.

## Data Availability

All data that support the findings of the present study are available from the corresponding author through reasonable request.
