# Supplementary material for: Socioeconomic status impacts cognitive and socioemotional processes in healthy ageing
Source: Sci Rep. 2022 Apr 11;12:6048. doi: 10.1038/s41598-022-09580-4 (PMC9001669; doi:10.1038/s41598-022-09580-4)
Supplement: Supplementary file 1 — Supplementary Information. [file 41598_2022_9580_MOESM1_ESM.pdf]

Supplementary material

Socioeconomic status impacts cognitive and socioemotional processes in healthy ageing

Joaquín Migeot[1,2], Mariela Calivar[3], Hugo Granchetti[4], Agustín Ibáñez[2,5,6,7], & Sol Fittipaldi[2,5,6,8]*

[1] Center for Social and Cognitive Neuroscience (CSCN), School of Psychology, Universidad Adolfo Ibáñez, Santiago de Chile, Chile.
[2] Latin American Brain Health Institute (BrainLat), Universidad Adolfo Ibáñez, Santiago de Chile, Chile
[3] Centro de atención Primaria de la salud Zonda, Ministerio de Salud Pública de la Provincia de San Juan, Argentina
[4] Facultad de Farmacia y Bioquímica, Universidad de Buenos Aires, Argentina
[5] Cognitive Neuroscience Center (CNC), Universidad de San Andrés, Buenos Aires, Argentina
[6] National Scientific and Technical Research Council (CONICET), Buenos Aires, Argentina
[7] Global Brain Health Institute, University of California, San Francisco, USA & Trinity College Dublin (TCD), Ireland
[8] Facultad de Psicología, Universidad Nacional de Córdoba, Córdoba, Argentina

*Corresponding author: Sol Fittipaldi
Cognitive Neuroscience Center (CNC), Universidad de San Andres
Vito Dumas 284, B1644BID Victoria, Argentina, Tel. +54-11-4725-7000, http://udesa.edu.ar/cnc, sfittipaldi@udesa.edu.ar

1. Requirements for path analysis models

a) Sample size: Since our sample size (*n* = 58) marginally meets the requirement of being ten times the number of paths included in the models (*q* = 6)[1,2], we employed a data imputation strategy to handle missing data (**Supplementary material 3**).

b) Directionality: The causal relationships established in the models (i.e., SES → cognitive state/executive functions → social cognition → social emotions [model 1: envy; model 2: *Schadenfreude*]) are based on an extensive literature review (as described in the **Introduction**). In addition, the causal effect of cognitive processes over socioemotional ones has been previously reported in structural equation and path analysis models[3-5].

c) Score reliability: Total score in the Addenbrooke's Cognitive Examination – III (ACE-III), the INECO Frontal Screening (IFS), the Mini-Social Cognition and Emotional Assessment (Mini-SEA), and the envy and *Schadenfreude* task were employed to measure cognitive state, executive functions, social cognition, and social emotions (i.e., envy and *Schadenfreude*), respectively. The ACE-III shows excellent internal consistency (Cronbach's α coefficient = 0.88)[6] and the IFS shows very good internal consistency (Cronbach's alpha = .80)[7]. For its part, the Mini-SEA has been successfully employed to assess social cognition in older adults[8,9], showing very good sensitivity and specificity in differentiating those with and without dementia[8,10,11]. Lastly, the envy and *Schadenfreude* task has been previously validated in older adults, producing the expected effects, and capturing its canonical neuroanatomical correlates[12,13].

d) Normality of the variables: Shapiro-Wilk's tests[14] and visual inspection of normal Q-Q plots were performed to test whether cognitive state, executive functions, social cognition, and social emotions (i.e., envy and *Schadenfreude*) scores approximated normal distribution within each group included in the models (i.e., low-SES, medium-low-SES, medium-high-SES, and high-SES groups – **Supplementary material 2**). Results revealed that all variables except executive functions' score in the medium-low-SES group met this requirement (**Supplementary Table S20**).

2. Quadripartite group formation strategy

To increase data variability in the implementation of path analysis and subsequent linear regression analyses, B level was considered as the high-SES group (*n* = 15), C_a as the medium-high-SES group (*n* = 13), D as the medium-low-SES group (*n* = 18), and E as the low-SES group (*n* = 12) (**Supplementary Table S2**).

3. Data imputation

To prevent a 21% sample loss (12 participants) by removing participants with missing data on at least one variable (**Supplementary Table S3**), we employed a data imputation by

regression method. This strategy consists of performing a linear regression to predict missing values for each participant as a linear combination of the remaining observed values[15].

4. Exploratory analyses with cognitive subdomains

For exploratory proposes, we ran five path analysis models (one for each cognitive subdomain derived from ACE-III instead of the total score) for each social emotion (envy and *Schadenfreude*). Results revealed that the ten models showed good fit indexes (**Supplementary Tables S8** and **S9**), supporting the inclusion of a single cognitive supradomain in our main models represented by ACE-III total score. Executive functions consistently predicted social cognition in all models (**Supplementary Tables S10** to **S19**, and **Fig. S1** and **S2**), reflecting the robustness of this relationship. Relatedly, verbal fluency significantly predicted social cognition (**Supplementary Tables S12** and **S17**). Verbal fluency is closed linked to cognitive flexibility[16,17], an executive function necessary for complex social cognition (e.g., perspective taking and theory of mind)[18,19]. Language also predicted social cognition (**Supplementary Tables S13** and **S18**), which is expected given language comprehension demands of the Faux-Pas (theory of mind) subtest of the Mini-SEA, which comprises text-based scenarios[20,21]. For its part, orientation and attention (**Supplementary Tables S10** and **S15**), memory (**Supplementary Tables S11** and **S16**), and visuospatial skills (**Supplementary Tables S14** and **S19**) did not predict social cognition. Arguably, these more basic cognitive domains are less directly related to social cognition processes[22]. In support of this interpretation, correlational evidence from neurodegenerative conditions shows a selective relationship of verbal fluency[23] and language comprehension[20] with social cognition.

Supplementary tables

Table S1. ESOMAR dimensions among groups

ESOMAR dimension	Low -SES	High-SES	Statistics
Educational level			
Incomplete primary school	5	0	$\chi 2(4, n = 58) = 45.56$, $p < 0.001$
Primary school	21	0	
High school	3	11	
Technical studies	1	3	
University studies	0	14	
Occupation			
Occasional worker	9	0	$\chi 2(5, n = 40) = 40.00$, $p < 0.001$
Non-qualified worker	9	0	
Qualified worker	3	0	
Low-, middle-level administrative worker	0	4	
Middle-level executive worker	0	14	
Senior-level executive worker	0	1	

Categorical variables were analyzed with Chi-squared test. SES: socioeconomic status.

Table S2. Demographic data corresponding to the quadripartite group formation strategy

Variable	Low-SES (n = 12)	Medium-low-SES (n = 18)	Medium-high-SES (n = 13)	High-SES (n = 15)	Statistics
Sex					
F	5	10	6	10	$\chi^2(3, n = 58) = 2.03, p = 0.57$
M	7	8	7	5	
Years of education	5.33 (2.23)	8.44 (2.64)	12.85 (1.95)	18.07 (2.16)	$F(3, 54) = 81.95$, $p < 0.001$, $\eta^2 = 0.82$*
Occupational status					
Active	10	11	6	13	$\chi^2(3, n = 58) = 7.03, p = 0.07$
Retired	2	7	7	2	
Dominant hand					
Right	11	17	13	13	$\chi^2(3, n = 58) = 2.02, p = 0.57$

Left	1	1	0	2	
Age	60.67 (6.76)	65.61 (7.66)	66.00 (7.96)	62.13 (7.07)	$F(3, 54) = 1.71$, $p = 0.18$, $\eta^2 = 0.09$
Depressive symptoms (BDI-II score)	16.00 (13.87)	9.50 (8.18)	5.83 (4.11)	7.29 (6.73)	$F(3, 48) = 2.74$, $p = 0.05$, $\eta^2 = 0.15$

Data are presented as mean (*SD*) except for sex, occupational status, and handedness. Chi square tests were applied to analyze categorical variables. ANOVA tests were employed to analyze continuous variables. BDI-II: Beck Depression Inventory-II; SES: socioeconomic status. *Bonferroni *post hoc* analysis showed that all groups differed from each other.

Table S3. Number of data points per variable

**Variable**	**Low-SES (*n* = 30)**	**High-SES (*n* = 28)**
Depressive symptoms (BDI-II score)	26	26
Cognitive state (ACE-III total score)	30	28
Executive functions (IFS total score)	30	23
Social cognition (Mini-SEA total score)	24	27
Social emotion: envy weighted score	30	28
Social emotion: s*chadenfreude* weighted score	30	28

ACE-III: Addenbrooke's Cognitive Examination - III; BDI-II: Beck Depression Inventory-II; IFS: INECO Frontal Screening; Mini-SEA: Mini-Social Cognition and Emotional Assessment; SES: socioeconomic status.

Table S4. Path coefficients for the envy model

**Effects**	**Non standardized**		***p*-value**	**Standardized**	
	**Coef**	**SE**		**Coef**	**SE**
SES → Cognitive state	5.66	0.75	< 0.001	0.71	0.06
SES → Executive functions	0.68	0.40	0.10	0.21	0.15

Cognitive state → Executive functions	0.24	0.05	< 0.001	0.59	0.13
Cognitive state → Social cognition	0.10	0.04	0.01	0.32	0.15
Executive functions → Social cognition	0.37	0.10	< 0.001	0.48	0.14
Social cognition → Social emotion: envy	0.28	0.91	0.01	0.37	0.12
SES → Cognitive state → Social cognition	0.57	0.27	0.02	0.23	0.24
SES → Executive functions → Social cognition	0.25	0.20	0.15	0.10	0.16
SES → Cognitive state → Executive functions → Social cognition	0.51	0.19	< 0.001	0.20	0.19
SES total effect on social emotion: envy	0.37	0.13	0.01	0.20	0.06

SES: socioeconomic status.

Table S5. Confidence intervals of path coefficients at 95% and 97.5% for envy model

Effects	Lower 2.5%	Lower 5%	Estimate	Upper 5%	Upper 2.5%
SES → Cognitive state	3.92	4.12	5.66	7.15	7.37
SES → Executive functions	-0.47	-0.30	0.67	1.45	1.57
Cognitive state → Executive functions	0.09	0.11	0.24	0.40	0.42
Cognitive state → Social cognition	0.01	0.02	0.10	0.19	0.21
Executive functions → Social cognition	0.13	0.16	0.37	0.63	0.68
Social cognition → Social emotion: envy	0.07	0.09	0.27	0.50	0.53

SES → Cognitive state → Social cognition	0.02	0.09	0.57	1.19	1.29
SES → Executive functions → Social cognition	-0.14	-0.08	0.25	0.72	0.80
SES → Cognitive state → Executive functions → Social cognition	0.19	0.22	0.51	1.03	1.12
SES total effect on social emotion: envy	0.12	0.15	0.37	0.64	0.69

Confidence intervals were calculated through 10,000 bootstrap iterations as per Mallinckrodt et al.[24]). SES: socioeconomic status.

Table S6. Path coefficients for the *Schadenfreude* model

**Effects**	**Non standardized**		***p*-value**	**Standardized**	
	**Coef**	**SE**		**Coef**	**SE**
SES → Cognitive state	5.66	0.75	< 0.001	0.71	0.06
SES → Executive functions	0.68	0.40	0.10	0.21	0.15
Cognitive state → Executive functions	0.24	0.05	< 0.001	0.59	0.13
Cognitive state → Social cognition	0.10	0.04	0.01	0.32	0.15
Executive functions → Social cognition	0.37	0.10	< 0.001	0.48	0.14
Social cognition → Social emotion: *Schadenfreude*	0.14	0.15	0.32	0.13	0.13
SES → Cognitive state → Social cognition	0.57	0.27	0.02	0.23	0.24
SES → Executive functions → Social cognition	0.25	0.20	0.15	0.10	0.16
SES → Cognitive state → Executive functions → Social cognition	0.51	0.19	< 0.001	0.20	0.19

SES total effect on social emotion: *Schadenfreude*	0.19	0.22	0.34	0.07	0.08

SES: socioeconomic status.

Table S7. Confidence intervals of path coefficients at 95% and 97.5% for *Schadenfreude* model

Effects	Lower 2.5%	Lower 5%	Estimate	Upper 5%	Upper 2.5%
SES → Cognitive state	3.92	4.12	5.66	7.15	7.37
SES → Executive functions	-0.47	-0.30	0.67	1.45	1.57
Cognitive state → Executive functions	0.09	0.11	0.24	0.40	0.42
Cognitive state → Social cognition	0.01	0.02	0.10	0.19	0.21
Executive functions → Social cognition	0.13	0.16	0.37	0.63	0.68
Social cognition → Social emotion: Schadenfreude	-0.19	-0.15	0.14	0.46	0.51
SES → Cognitive state → Social cognition	0.02	0.09	0.57	1.19	1.29
SES → Executive functions → Social cognition	-0.14	-0.08	0.25	0.12	0.80
SES → Cognitive state → Executive functions → Social cognition	0.19	0.22	0.51	1.03	1.12
SES total effect on social emotion: Schadenfreude	-0.23	-0.19	0.19	0.66	0.73

Confidence intervals were calculated through 10,000 bootstrap iterations as per Mallinckrodt et al.[24]). SES: socioeconomic status.

Table S8. Fit indexes for models with cognitive state subdomains including envy as final outcome

**Model**	**Chi-square**	***p*-value**	**RMSEA**	**SRMR**	**NFI**	**GFI**	**CFI**

Orientation and attention	1.64	0.80	0.00 (0.00 - 0.13)	0.03	0.98	0.99	1.00
Memory	4.12	0.39	0.02 (0.00 – 0.20)	0.05	0.96	0.97	1.00
Verbal fluency	5.48	0.24	0.08 (0.00 – 0.23)	0.05	0.95	0.96	0.99
Language	6.13	0.19	0.10 (0.00 – 0.24)	0.05	0.95	0.96	0.98
Visuospatial skills	6.56	0.16	0.11 (0.00 – 0.25)	0.07	0.94	0.96	0.97

Table S9. Fit indexes for models with cognitive state subdomains including *Schadenfreude* as final outcome

**Model**	**Chi-square**	***p*-value**	**RMSEA**	**SRMR**	**NFI**	**GFI**	**CFI**
Orientation and attention	7.40	0.12	0.12	0.07	0.93	0.96	0.96
Memory	9.91	0.04	0.16	0.10	0.91	0.94	0.94
Verbal fluency	6.68	0.14	0.11	0.07	0.94	0.96	0.97
Language	8.04	0.09	0.13	0.09	0.94	0.95	0.97
Visuospatial skills	8.21	0.08	0.14	0.08	0.92	0.95	0.95

Table S10. Path coefficients for the orientation and attention model including envy as final outcome

**Effects**	**Non standardized**		***p*-value**	**Standardized**	
	**Coef**	**SE**		**Coef**	**SE**
SES → Orientation and attention	0.79 (0.49 – 1.05)	0.16	> 0.001	0.56	0.09
SES → Executive functions	1.65 (0.98 – 2.32)	0.40	> 0.001	0.51	0.12
Orientation and attention → Executive functions	0.48 (-0.12 – 1.19)	0.28	0.09	0.21	0.12

Orientation and attention → Social cognition	0.23 (-0.09 – 0.65)	0.19	0.22	0.13	0.10
Executive functions → Social cognition	0.51 (0.34 – 0.73)	0.08	> 0.001	0.65	0.08
Social cognition → Social emotion: envy	0.28 (0.09 – 0.50)	0.09	0.01	0.37	0.12
SES → Orientation and attention → Social cognition	0.18 (-0.06 – 0.56)	0.15	0.14	0.07	0.15
SES → Executive functions → Social cognition	0.84 (0.43 – 1.37)	0.24	> 0.001	0.33	0.24
SES → Orientation and attention → Executive functions → Social cognition	0.19 (-0.03 – 0.46)	0.12	0.08	0.08	0.12
SES total effect on social emotion: envy	0.33 (0.14 – 0.58)	0.11	0.001	0.18	0.06

Non-standardized estimates are presented as coefficient (lower – upper 95% confidence intervals, obtained through 10,000 bootstrap iterations as per Mallinckrodt et al.[24]). SES: socioeconomic status.

Table S11. Path coefficients for the memory model including envy as final outcome

**Effects**	**Non standardized**		***p*-value**	**Standardized**	
	**Coef**	**SE**		**Coef**	**SE**
SES → Memory	1.67 (1.07 – 2.40)	0.37	> 0.001	0.51	0.07
SES → Executive functions	1.51 (0.81 – 2.23)	0.37	> 0.001	0.47	0.12
Memory → Executive functions	0.31 (0.09 – 0.62)	0.11	0.01	0.31	0.11
Memory → Social cognition	0.03 (-0.13 – 0.19)	0.09	0.77	0.03	0.10
Executive functions → Social cognition	0.54 (0.36 – 0.75)	0.09	> 0.001	0.70	0.09
Social cognition → Social emotion: envy	0.28 (0.09 – 0.50)	0.09	0.01	0.37	0.12

SES → Memory → Social cognition	0.04 (-0.20 - 0.32)	0.13	0.71	0.02	0.14
SES → Executive functions → Social cognition	0.82 (0.34 - 1.36)	0.27	> 0.001	0.33	0.24
SES → Memory → Executive functions → Social cognition	0.28 (0.11 - 0.54)	0.11	0.01	0.11	0.13
SES total effect on social emotion: envy	0.31 (0.13 – 0.54)	0.11	0.01	0.17	0.05

Non-standardized estimates are presented as coefficient (lower – upper 95% confidence intervals, obtained through 10,000 bootstrap iterations as per Mallinckrodt et al.[24]). SES: socioeconomic status.

Table S12. Path coefficients for the verbal fluency model including envy as final outcome

Effects	Non standardized		*p*-value	Standardized	
	Coef	SE		Coef	SE
SES → Verbal fluency	1.13 (0.63 – 1.62)	0.25	> 0.001	0.52	0.25
SES → Executive functions	1.43 (0.74 – 2.12)	0.36	> 0.001	0.44	0.35
Verbal fluency → Executive functions	0.53 (0.10 – 1.00)	0.16	0,001	0.36	0.23
Verbal fluency → Social cognition	0.26 (-0.03 – 0.51)	0.13	0,04	0.22	0.14
Executive functions → Social cognition	0.46 (0.27 – 0.71)	0.09	> 0.001	0.59	0.11
Social cognition → Social emotion: envy	0.28 (0.10 – 0.51)	0.09	0,01	0.37	0.11
SES → Verbal fluency → Social cognition	0.29 (-0.04 – 0.64)	0.17	0.08	0.11	0.16
SES → Executive functions → Social cognition	0.65 (0.26 – 1.23)	0.25	> 0.001	0.26	0.20

SES → Verbal fluency → Executive functions → Social cognition	0.27 (0.06 – 0.58)	0.13	0.02	0.11	0.12
SES total effect on social emotion: envy	0.33 (0.13 – 0.59)	0.12	0.001	0.18	0.06

Non-standardized estimates are presented as coefficient (lower – upper 95% confidence intervals, obtained through 10,000 bootstrap iterations as per Mallinckrodt et al.[24]). SES: socioeconomic status.

Table S13. Path coefficients for the language model including envy as final outcome

**Effects**	**Non standardized**		***p*-value**	**Standardized**	
	**Coef**	**SE**		**Coef**	**SE**
SES → Language	1.56 (1.09 – 2.07)	0.23	> 0.001	0.67	0.06
SES → Executive functions	1.31 (0.45 – 2.16)	0.43	0.01	0.40	0.14
Language → Executive functions	0.46 (0.13 – 1.01)	0.18	0.01	0.33	0.12
Language → Social cognition	0.37 (0.12 – 0.63)	0.12	0.01	0.34	0.11
Executive functions → Social cognition	0.40 (0.24 – 0.62)	0.08	> 0.001	0.51	0.10
Social cognition → Social emotion: envy	0.28 (0.10 – 0.51)	0.09	0.01	0.37	0.12
SES → Language → Social cognition	0.57 (0.17 – 1.03)	0.22	0.01	0.23	0.20
SES → Executive functions → Social cognition	0.52 (1.16 – 0.97)	0.21	0.01	0.21	0.20
SES → Language → Executive functions → Social cognition	0.29 (0.09 – 0.58)	0.13	0.01	0.11	0.14
SES total effect on social emotion: envy	0.38 (0.15 – 0.64)	0.12	0.01	0.20	0.06

Non-standardized estimates are presented as coefficient (lower – upper 95% confidence intervals, obtained through 10,000 bootstrap iterations as per Mallinckrodt et al.[24]). SES: socioeconomic status.

Table S14. Path coefficients for the visuospatial skills model including envy as final outcome

Effects	Non standardized		*p*-value	Standardized	
	Coef	SE		Coef	SE
SES → Visuospatial skills	0.56 (0.23 – 0.92)	0.16	> 0.001	0.43	0.10
SES → Executive functions	1.58 (0.94 – 2.25)	0.35	> 0.001	0.49	0.10
Visuospatial skills → Executive functions	0.80 (0.26 – 1.37)	0.27	0.01	0.32	0.13
Visuospatial skills → Social cognition	0.21 (-0.19 – 0.56)	0.21	0.31	0.11	0.10
Executive functions → Social cognition	0.51 (0.35 – 0.76)	0.08	> 0.001	0.66	0.09
Social cognition → Social emotion: envy	0.28 (0.09 – 0.50)	0.09	0.01	0.37	0.12
SES → Visuospatial skills → Social cognition	0.12 (-0.07 - 0.43)	0.12	0.19	0.05	0.12
SES → Executive functions → Social cognition	0.81 (0.44 - 1.27)	0.21	> 0.001	0.32	0.22
SES → Visuospatial skills → Executive functions → Social cognition	0.23 (0.04 - 0.63)	0.15	0.01	0.09	0.11
SES total effect on social emotion: envy	0.32 (0.12 – 0.54)	0.11	0.01	0.17	0.05

Non-standardized estimates are presented as coefficient (lower – upper 95% confidence intervals, obtained through 10,000 bootstrap iterations as per Mallinckrodt et al.[24]). SES: socioeconomic status.

Table S15. Path coefficients for the orientation and attention model including *Schadenfreude* as final outcome

Effects	Non standardized		*p*-value	Standardized	
	Coef	SE		Coef	SE
SES → Orientation and attention	0.79 (0.49 – 1.05)	0.16	> 0.001	0.56	0.09
SES → Executive functions	1.65 (0.98 – 2.32)	0.40	> 0.001	0.51	0.12
Orientation and attention → Executive functions	0.48 (-0.12 – 1.19)	0.28	0.09	0.21	0.12
Orientation and attention → Social cognition	0.23 (-0.09 – 0.65)	0.19	0.22	0.13	0.10
Executive functions → Social cognition	0.51 (0.34 – 0.73)	0.08	> 0.001	0.65	0.08
Social cognition → Social emotion: *Schadenfreude*	0.14 (-0.15 – 0.47)	0.16	0.35	0.13	0.14
SES → Orientation and attention → Social cognition	0.18 (-0.06 – 0.56)	0.15	0.14	0.07	0.15
SES → Executive functions → Social cognition	0.84 (0.43 – 1.37)	0.24	> 0.001	0.33	0.24
SES → Orientation and attention → Executive functions → Social cognition	0.19 (-0.03 – 0.46)	0.12	0.08	0.08	0.12
SES total effect on social emotion: *Schadenfreude*	0.14 (-0.15 – 0.47)	0.16	0.35	0.13	0.14

Non-standardized estimates are presented as coefficient (lower – upper 95% confidence intervals, obtained through 10,000 bootstrap iterations as per Mallinckrodt et al.[24]). SES: socioeconomic status.

Table S16. Path coefficients for the memory model including *Schadenfreude* as final outcome

Effects	Non standardized		*p*-value	Standardized	
	Coef	SE		Coef	SE
SES → Memory	1.67 (1.07 – 2.40)	0.37	> 0.001	0.51	0.07

SES → Executive functions	1.51 (0.81 – 2.23)	0.37	> 0.001	0.47	0.12
Memory → Executive functions	0.31 (0.09 – 0.62)	0.11	0.01	0.31	0.11
Memory → Social cognition	0.03 (-0.13 – 0.19)	0.09	0.77	0.03	0.10
Executive functions → Social cognition	0.54 (0.36 – 0.75)	0.09	> 0.001	0.70	0.09
Social cognition → Social emotion: *Schadenfreude*	0.14 (-0.15 – 0.47)	0.16	0.35	0.13	0.14
SES → Memory → Social cognition	0.04 (-0.20 - 0.32)	0.13	0.71	0.02	0.14
SES → Executive functions → Social cognition	0.82 (0.34 - 1.36)	0.27	> 0.001	0.33	0.24
SES → Memory → Executive functions → Social cognition	0.28 (0.11 - 0.54)	0.11	0.01	0.11	0.13
SES total effect on social emotion: *Schadenfreude*	0.14 (-0.15 – 0.47)	0.16	0.35	0.13	0.14

Non-standardized estimates are presented as coefficient (lower – upper 95% confidence intervals, obtained through 10,000 bootstrap iterations as per Mallinckrodt et al.[24]). SES: socioeconomic status.

Table S17. Path coefficients for the verbal fluency model including *Schadenfreude* as final outcome

**Effects**	**Non standardized**		***p*-value**	**Standardized**	
	**Coef**	**SE**		**Coef**	**SE**
SES → Verbal fluency	1.13 (0.63 – 1.62)	0.25	> 0.001	0.52	0.25
SES → Executive functions	1.43 (0.74 – 2.12)	0.36	> 0.001	0.44	0.35
Verbal fluency → Executive functions	0.53 (0.10 – 1.00)	0.16	0,001	0.36	0.23
Verbal fluency → Social cognition	0.26 (-0.03 – 0.51)	0.13	0,04	0.22	0.14

Executive functions → Social cognition	0.46 (0.27 – 0.71)	0.09	> 0.001	0.59	0.11
Social cognition → Social emotion: *Schadenfreude*	0.14 (-0.15 – 0.47)	0.16	0.35	0.13	0.14
SES → Verbal fluency → Social cognition	0.29 (-0.04 – 0.64)	0.17	0.08	0.11	0.16
SES → Executive functions → Social cognition	0.65 (0.26 – 1.23)	0.25	> 0.001	0.26	0.20
SES → Verbal fluency → Executive functions → Social cognition	0.27 (0.06 – 0.58)	0.13	0.02	0.11	0.12
SES total effect on social emotion: *Schadenfreude*	0.14 (-0.15 – 0.47)	0.16	0.35	0.13	0.14

Non-standardized estimates are presented as coefficient (lower – upper 95% confidence intervals, obtained through 10,000 bootstrap iterations as per Mallinckrodt et al.[24]). SES: socioeconomic status.

Table S18. Path coefficients of the language model including *Schadenfreude* as final outcome

Effects	Non standardized		*p*-value	Standardized	
	Coef	SE		Coef	SE
SES → Language	1.56 (1.09 – 2.07)	0.23	> 0.001	0.67	0.06
SES → Executive functions	1.31 (0.45 – 2.16)	0.43	0.01	0.40	0.14
Language → Executive functions	0.46 (0.13 – 1.01)	0.18	0.01	0.33	0.12
Language → Social cognition	0.37 (0.12 – 0.63)	0.12	0.01	0.34	0.11
Executive functions → Social cognition	0.40 (0.24 – 0.62)	0.08	> 0.001	0.51	0.10
Social cognition → Social emotion: *Schadenfreude*	0.14 (-0.15 – 0.47)	0.16	0.35	0.13	0.14
SES → Language → Social cognition	0.57 (0.17 – 1.03)	0.22	0.01	0.23	0.20

SES → Executive functions → Social cognition	0.52 (1.16 – 0.97)	0.21	0.01	0.21	0.20
SES → Language → Executive functions → Social cognition	0.29 (0.09 – 0.58)	0.13	0.01	0.11	0.14
SES total effect on social emotion: *Schadenfreude*	0.14 (-0.15 – 0.47)	0.16	0.35	0.13	0.14

Non-standardized estimates are presented as coefficient (lower – upper 95% confidence intervals, obtained through 10,000 bootstrap iterations as per Mallinckrodt et al.[24]). SES: socioeconomic status.

Table S19. Path coefficients of the visuospatial skills model including *Schadenfreude* as final outcome

**Effects**	**Non standardized**		***p*-value**	**Standardized**	
	**Coef**	**SE**		**Coef**	**SE**
SES → Visuospatial skills	0.56 (0.23 – 0.92)	0.16	> 0.001	0.43	0.10
SES → Executive functions	1.58 (0.94 – 2.25)	0.35	> 0.001	0.49	0.10
Visuospatial skills → Executive functions	0.80 (0.26 – 1.37)	0.27	0.01	0.32	0.13
Visuospatial skills → Social cognition	0.21 (-0.19 – 0.56)	0.21	0.31	0.11	0.10
Executive functions → Social cognition	0.51 (0.35 – 0.76)	0.08	> 0.001	0.66	0.09
Social cognition → Social emotion: *Schadenfreude*	0.14 (-0.15 – 0.47)	0.16	0.35	0.13	0.14
SES → Visuospatial skills → Social cognition	0.12 (-0.07 - 0.43)	0.12	0.19	0.05	0.12
SES → Executive functions → Social cognition	0.81 (0.44 - 1.27)	0.21	> 0.001	0.32	0.22
SES → Visuospatial skills → Executive functions → Social cognition	0.23 (0.04 - 0.63)	0.15	0.01	0.09	0.11

SES total effect on social emotion: *Schadenfreude*	0.14 (-0.15 – 0.47)	0.16	0.35	0.13	0.14

Non-standardized estimates are presented as coefficient (lower – upper 95% confidence intervals, obtained through 10,000 bootstrap iterations as per Mallinckrodt et al.[24]). SES: socioeconomic status.

Table S20. Shapiro-Wilk' tests for variables included in the models

Variable	Group	Statistic	df	*p*-value*
Cognitive state (ACE-III total score)	Low-SES	0.93	12	> 0.99
	Medium-low-SES	0.96	18	> 0.99
	Medium-high-SES	0.97	13	> 0.99
	High-SES	0.94	15	> 0.99
Executive functions (IFS total score)	Low-SES	0.96	12	> 0.99
	Medium-low-SES	0.74	18	0.01
	Medium-high-SES	0.93	13	> 0.99
	High-SES	0.99	15	> 0.99
Social cognition (Mini-SEA total score)	Low-SES	0.88	12	0.48
	Medium-low-SES	0.97	18	> 0.99
	Medium-high-SES	0.94	13	> 0.99
	High-SES	0.95	15	> 0.99
Social emotion: envy	Low-SES	0.89	12	0.14
	Medium-low-SES	0.89	18	0.28
	Medium-high-SES	0.82	13	0.72

	High-SES	0.96	15	> 0.99
Social emotion: *Schadenfreude*	Low-SES	0.97	12	> 0.99
	Medium-low-SES	0.97	18	> 0.99
	Medium-high-SES	0.94	13	> 0.99
	High-SES	0.91	15	0.34

**p*-values are Bonferroni-corrected for 4 tests. ACE-III: Addenbrooke's Cognitive Examination - III; BDI-II: Beck Depression Inventory-II; IFS: INECO Frontal Screening; Mini-SEA: Mini-Social Cognition and Emotional Assessment; SES: socioeconomic status.

Supplementary figures

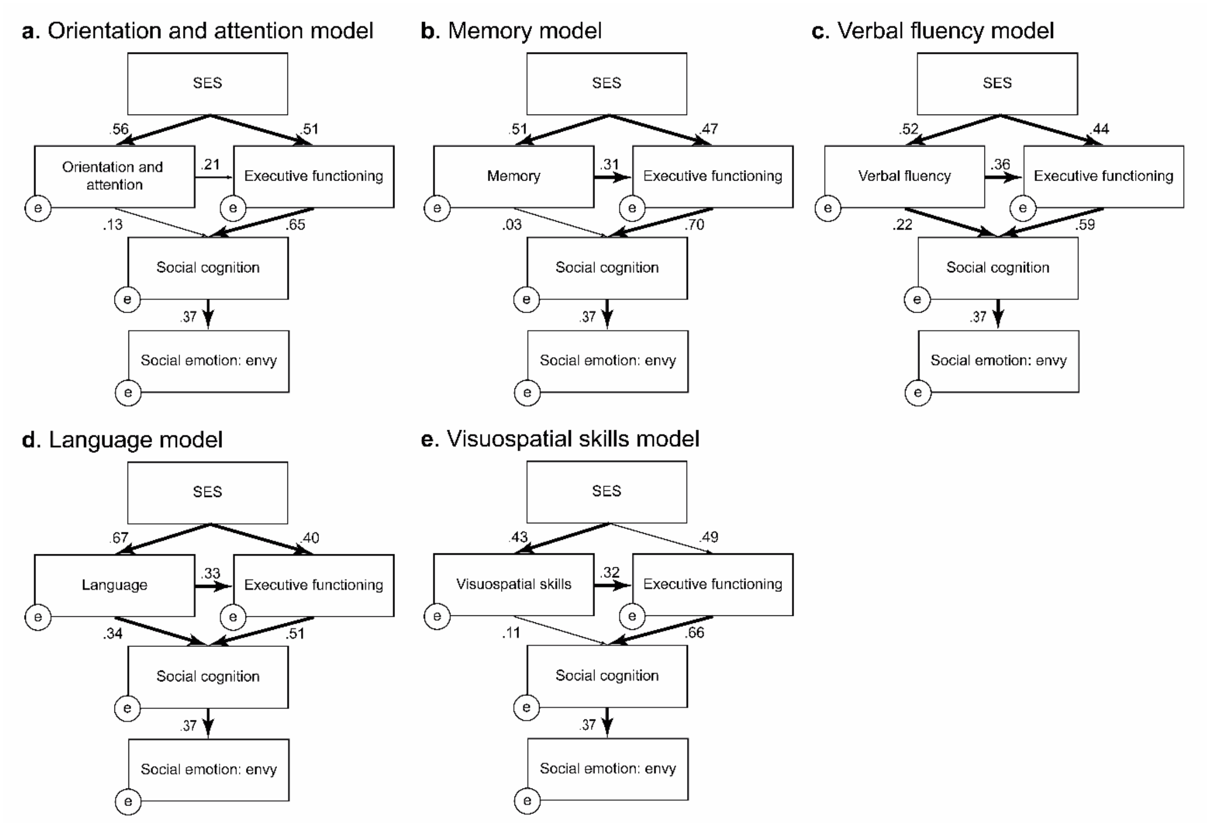

Figure S1. Exploratory models including envy as final outcome. Path analysis models and standardized path coefficients weights including **a.** orientation and attention, **b.** memory, **c.** verbal fluency, **d.** language, and **e.** visuospatial skills as cognitive state subdomains and envy as outcome social emotion. Bold arrows depict statistically significant paths. SES: socioeconomic status.

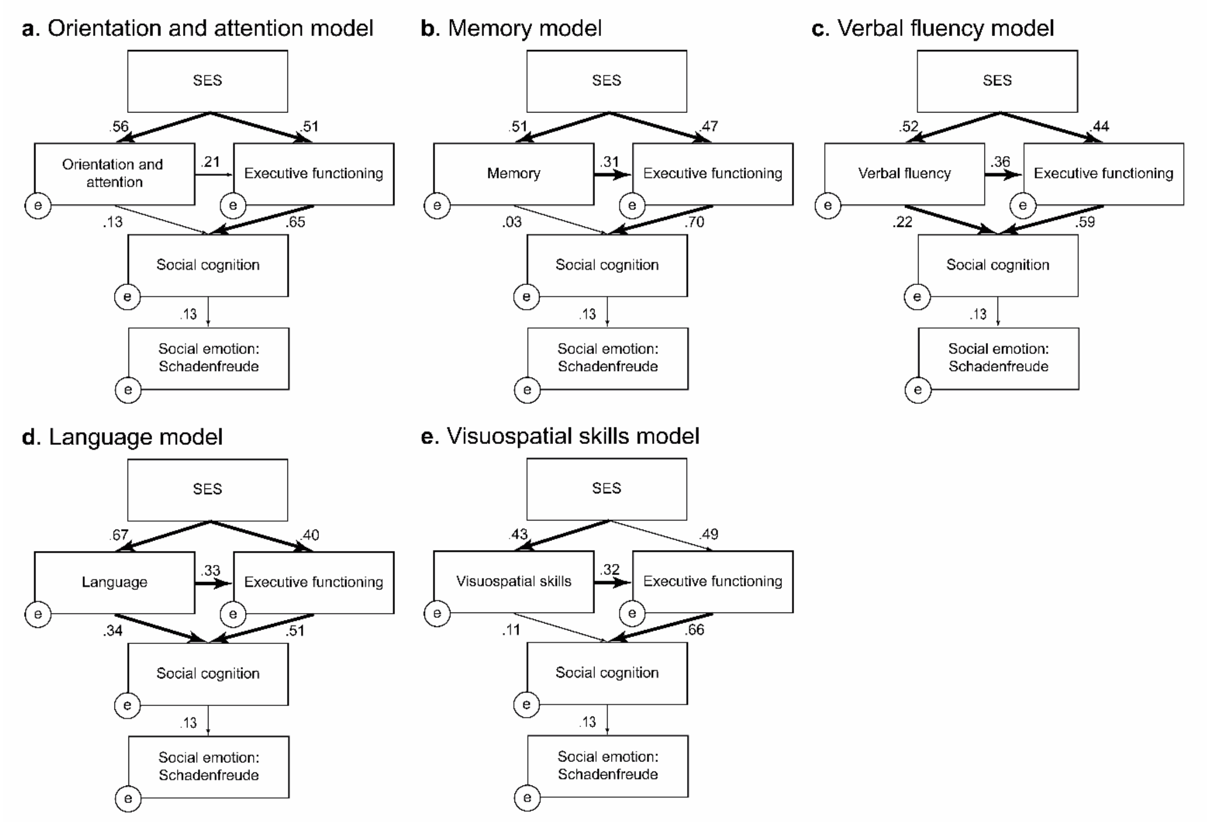

Figure S2. Exploratory models including *Schadenfreude* as final outcome. Path analysis models and standardized path coefficients weights including **a.** orientation and attention, **b.** memory, **c.** verbal fluency, **d.** language, and **e.** visuospatial skills as cognitive state subdomains and *Schadenfreude* as outcome social emotion. Bold arrows depict statistically significant paths. SES: socioeconomic status.

Supplementary references

1 Kline, R. B. *Principles and practice of structural equation modeling.* (Guilford publications, 2015).

2 Jackson, D. L. Revisiting sample size and number of parameter estimates: Some support for the N:q hypothesis. *Structural Equation Modeling* **10**, 128-141; 10.1207/S15328007SEM1001_6 (2003).

3 Schmidt, S. J., Mueller, D. R. & Roder, V. Social Cognition as a Mediator Variable Between Neurocognition and Functional Outcome in Schizophrenia: Empirical Review and New Results by Structural Equation Modeling. *Schizophrenia Bulletin* **37**, S41-S54; 10.1093/schbul/sbr079 (2011).

4 Ibanez, A. *et al.* Empathy, sex and fluid intelligence as predictors of theory of mind. *Personality and Individual Differences* **54**, 616-621; 10.1016/j.paid.2012.11.022 (2013).

5 Ramírez-Luzuriaga, M. J. *et al.* Influence of enhanced nutrition and psychosocial stimulation in early childhood on cognitive functioning and psychological well-being in Guatemalan adults. *Social Science & Medicine* **275**, 113810; 10.1016/j.socscimed.2021.113810 (2021).

6 Hsieh, S., Schubert, S., Hoon, C., Mioshi, E. & Hodges, J. R. Validation of the Addenbrooke's Cognitive Examination III in frontotemporal dementia and Alzheimer's disease. *Dementia and geriatric cognitive disorders* **36**, 242-250; 10.1159/000351671 (2013).

7 Torralva, T., Roca, M., Gleichgerrcht, E., Lopez, P. & Manes, F. INECO Frontal Screening (IFS): a brief, sensitive, and specific tool to assess executive functions in dementia. *J Int Neuropsychol Soc* **15**, 777-786; 10.1017/S1355617709990415 (2009).

8 Bertoux, M. *et al.* Social cognition and emotional assessment (SEA) is a marker of medial and orbital frontal functions: A voxel-based morphometry study in behavioral variant of frontotemporal degeneration. *Journal of the International Neuropsychological Society* **18**, 972-985; 10.1017/S1355617712001300 (2012).

9 Funkiewiez, A., Bertoux, M., de Souza, L. C., Lévy, R. & Dubois, B. The SEA (social cognition and emotional assessment): a clinical neuropsychological tool for early diagnosis of frontal variant of frontotemporal lobar degeneration. *Neuropsychology* **26**, 81; 10.1037/a0025318 (2012).

10 Bertoux, M. *et al.* Neural correlates of the mini-SEA (Social cognition and Emotional Assessment) in behavioral variant frontotemporal dementia. *Brain Imaging and Behavior* **8**, 1-6; 10.1007/s11682-013-9261-0 (2014).

11 Bertoux, M., Funkiewiez, A., O'Callaghan, C., Dubois, B. & Hornberger, M. Sensitivity and specificity of ventromedial prefrontal cortex tests in behavioral variant frontotemporal dementia. *Alzheimer's and Dementia* **9**, S84-S94; 10.1016/j.jalz.2012.09.010 (2013).

12 Santamaría-García, H. *et al.* A lesion model of envy and Schadenfreude: legal, deservingness and moral dimensions as revealed by neurodegeneration. *Brain* **140**, 3357-3377; 10.1093/brain/awx269 (2017).

13 Baez, S. *et al.* Classifying Parkinson’s Disease Patients With Syntactic and Socio-emotional Verbal Measures. **12**; 10.3389/fnagi.2020.586233 (2020).

14 Shapiro, S. S. & Wilk, M. B. An Analysis of Variance Test for Normality (Complete Samples). *Biometrika* **52**, 591-611; 10.2307/2333709 (1965).

15 Blunch, N. Incomplete and Non-Normal Data in *Introduction to structural equation modeling using IBM SPSS statistics and AMOS* 217-234 (Sage, 2012).
16 Amunts, J. *et al.* Comprehensive verbal fluency features predict executive function performance. *Scientific Reports* **11**, 6929; 10.1038/s41598-021-85981-1 (2021).
17 Hedman, E., Hartelius, L. & Saldert, C. Word-finding difficulties in Parkinson's disease: Complex verbal fluency, executive functions and other influencing factors. *International Journal of Language & Communication Disorders*; 10.1111/1460-6984.12707 (2022).
18 Whitton, A. E., Henry, J. D. & Grisham, J. R. Moral rigidity in obsessive-compulsive disorder: Do abnormalities in inhibitory control, cognitive flexibility and disgust play a role? *Journal of Behavior Therapy and Experimental Psychiatry* **45**, 152-159; doi.org/10.1016/j.jbtep.2013.10.001 (2014).
19 Bock, A. M., Gallaway, K. C. & Hund, A. M. Specifying Links Between Executive Functioning and Theory of Mind during Middle Childhood: Cognitive Flexibility Predicts Social Understanding. *Journal of Cognition and Development* **16**, 509-521; 10.1080/15248372.2014.888350 (2015).
20 Chanial, C. *et al.* Assessment of implicit language and theory of mind in multiple sclerosis. *Annals of Physical and Rehabilitation Medicine* **63**, 111-115; 10.1016/j.rehab.2019.08.005 (2020).
21 Smogorzewska, J., Szumski, G. & Grygiel, P. Same or different? Theory of mind among children with and without disabilities. *PLOS ONE* **13**, e0202553; 10.1371/journal.pone.0202553 (2018).
22 Ibanez, A. *et al.* Social neuroscience: undoing the schism between neurology and psychiatry. *Soc Neurosci* **13**, 1-39; 10.1080/17470919.2016.1245214 (2018).
23 Torralva, T., Roca, M., Gleichgerrcht, E., Bekinschtein, T. & Manes, F. A neuropsychological battery to detect specific executive and social cognitive impairments in early frontotemporal dementia. *Brain* **132**, 1299-1309; 10.1093/brain/awp041 (2009).
24 Mallinckrodt, B., Abraham, W. T., Wei, M. & Russell, D. W. Advances in testing the statistical significance of mediation effects. *Journal of counseling psychology* **53**, 372; 10.1037/0022-0167.53.3.372 (2006).
